# Probing the structural evolution and electronic properties of divalent metal Be_2_Mg_*n*_ clusters from small to medium-size

**DOI:** 10.1038/s41598-020-63237-8

**Published:** 2020-04-08

**Authors:** Feige Zhang, Hairong Zhang, Wang Xin, Peng Chen, Yanfei Hu, Xiaoyi Zhang, Yaru Zhao

**Affiliations:** 1 School of Electrical and Electronic Engineering, Baoji University of Arts and Sciences, Baoji, 721016 China; 2 College of Physics and Optoelectronics Technology, Baoji University of Arts and Sciences, Baoji, 721016 China; 3 School of Physics and Electronic Engineering, Sichuan University of Science & Engineering, Zigong, 643000 China

**Keywords:** Electronic properties and materials, Chemical physics

## Abstract

Bimetallic clusters have aroused increased attention because of the ability to tune their own properties by changing size, shape, and doping. In present work, a structural search of the global minimum for divalent bimetal Be_2_Mg_*n*_ (*n* = 1–20) clusters are performed by utilizing CALYPSO structural searching method with subsequent DFT optimization. We investigate the evolution of geometries, electronic properties, and nature of bonding from small to medium-sized clusters. It is found that the structural transition from hollow 3D structures to filled cage-like frameworks emerges at *n* = 10 for Be_2_Mg_*n*_ clusters, which is obviously earlier than that of Mg_*n*_ clusters. The Be atoms prefer the surface sites in small cluster size, then one Be atom tend to embed itself inside the magnesium motif. At the number of Mg larger than eighteen, two Be atoms have been completely encapsulated by caged magnesium frameworks. In all Be_2_Mg_*n*_ clusters, the partial charge transfer from Mg to Be takes place. An increase in the occupations of the Be-2*p* and Mg-3*p* orbitals reveals the increasing metallic behavior of Be_2_Mg_*n*_ clusters. The analysis of stability shows that the cluster stability can be enhanced by Be atoms doping and the Be_2_Mg_8_ cluster possesses robust stability across the cluster size range of *n* = 1–20. There is *s*-*p* hybridization between the Be and Mg atoms leading to stronger Be-Mg bonds in Be_2_Mg_8_ cluster. This finding is supported by the multi-center bonds and Mayer bond order analysis.

## Introduction

Metallic clusters, in particular, have attracted a great deal of attention in recent years due to not only their unique properties of electronic, magnetic, optical, and mechanical but also the wide applications in nanomaterials, hydrogen storage, catalyst, biomedicine, and spintronics^1–7^. The typical metallic clusters include alkali metal clusters of Li_*n*_^8,9^ and Na_*n*_^10,11^, noble metal clusters of Au_*n*_^12–14^, Ag_*n*_^15,16^ and Cu_*n*_^17,18^, along with magnetic transition metal clusters of Fe_*n*_^19,20^, Co_*n*_^21,22^, Ni_*n*_^23–25^ and so on. Based on previous studies, one can draw the following conclusion that these clusters usually exhibit superior properties compared to metals in terms of activity, selectivity, stability, bonding character, and charge transfer. In addition, their superior properties sensitively depend on clusters size, element components, and dopants.

As a divalent metal, magnesium clusters have been extensively studied by both experimental and theoretical fields^26–31^. In previous works, one main focus of the studies is on size-induced nonmetal-to-metal transition of magnesium clusters. It is well known that the atom of divalent metal Mg has *s*^2^ closed-shell electron configuration as helium. So the bonding of the magnesium dimer is expected to be van der Waals-like^32^. As the number of atoms grows, the electronic properties of clusters transfer from van der Waals to covalent, then to metallic behavior. For example, Kaplan *et al*.^33^ point out that the binding in alkaline-earth trimers (Be_3_ and Mg_3_) has a mixed of physical (van der Walls) and chemical (nonadditive exchange) nature, which is different from their dimers. Gong *et al*.^34^ performed a detailed investigations in terms of the nearest neighbor bond length and *s-p* hybridization of the magnesium clusters. By measuring the photoelectron spectra (PES) of Mg_*n*_^‾^ (*n* = 2–22) clusters, Thomas and co-workers^35^ observed the *s-p* band gap of magnesium cluster anions to close at *n* = 18, signaling the onset of metallic behavior. Then the metallic behavior of Mg_*n*_^‾^ clusters was well explained by Jellinek and Acioli^36,37^ through an analysis of charged distribution characteristics of 3*p* orbital. In neutral Mg_*n*_ clusters, this nonmetal-to-metal transition has been reported to occur around *n* = 20^38^. Another main thrust of the studies is a systematic discussion on structural and energetic properties of magnesium clusters along with their magic number. Note that the Jellium model has been most successful for explaning the magic number of the simple alkali metal clusters, even for confirming the magic number of the noble metal Au_*n*_ clusters. For Mg_*n*_ clusters, the *ab initio* calculations indicate the magic number *N* = 4, 10, and 20 for small sized clusters^30,39–41^, which is in agreement with simple Jellium model. However, the finding of new experiment reveals the most intensive peaks in the mass spectra at *N* = 5, 10, 15, 18, and 20^31^. Thus one can find that the stability of the Mg_*n*_ clusters is not only related to their electronic shell configuration but also affected by geometric structures.

Despite a number of investigations have been carried out on the pure Mg_*n*_ clusters, however, little work has been done so far on the exploration of the divalent bimetal clusters. Beryllium, as one of the lightest divalent metal, has *s*^2^ valence electronic configuration. Therefore, the properties of the beryllium clusters are very similar to magnesium clusters. The small beryllium clusters also show van der Waals character bonding and the transition from nonmetal to metal occurs around Be_13_^42^. The theoretical studies indicate more stability of small sized beryllium clusters with magic number *N* = 4, 10, 17, and 20^43,44^. So it seems a systematic study is necessary to probe the structural evolution and bonding characters of Be-doped magnesium clusters. The starting point of this work can be summarized as follows: (1) We can find the most stable structures and elucidate the structural evolution process of Be_2_Mg_*n*_ (*n* = 1–20) clusters. (2) By discussing the bonding and electronic nature, we can gain a deeper understanding of the microscopic mechanisms of these bivalent bimetal clusters (3) It might provide some theoretical guidance for the development of the magnesium-based materials and their applications in catalyst, hydrogen storage, and corrosion-resistant.

## Results and discussions

### Geometric structure

**Figure 1:**
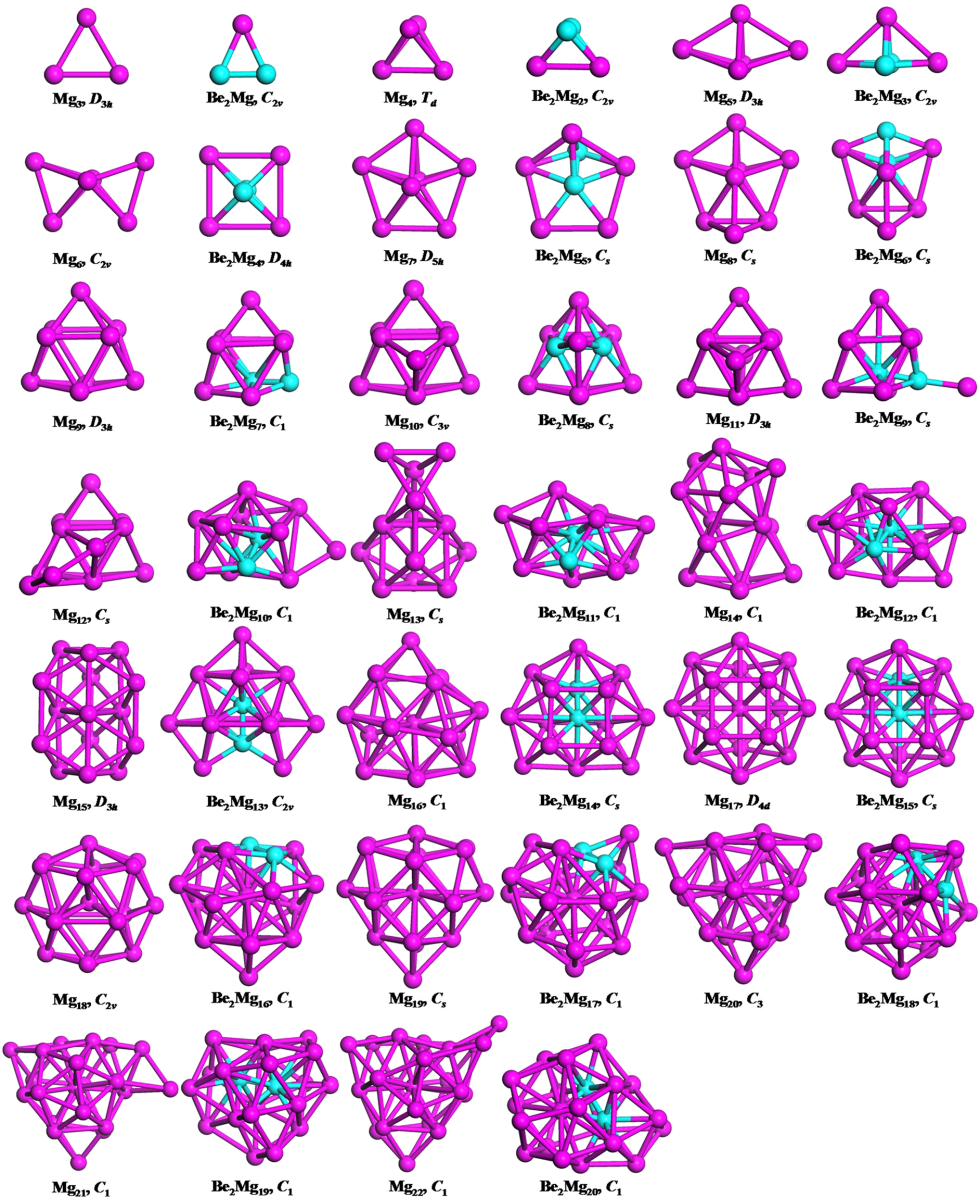
The most stable structures of the Mg_*n*+2_ and Be_2_Mg_*n*_ (*n* = 1–20) clusters,.

In light of their total energies of the low energetic and stable cluster candidates, the most stable isomers Be_2_Mg_*n*_ clusters in the size range of *n* = 1–20 are confirmed and presented in Fig. 1. Moreover, the typical low-lying isomers, together with their relative energies and symmetries of the Be_2_Mg_*n*_ clusters are shown in Figs. S1 and S2. (Supplementary Information). It can be noted that our theoretical dissociation energy (0.073 eV) for the Mg_2_ dimer agrees well with experimental result of 0.049 eV^36^. We can get the error in the calculation of the energy around 0.024 eV. It is clearly lower than the relative energies of the low-lying isomers. To discuss the effect of dopants on the geometries and properties of magnesium clusters, the most stable structures of pure Mg_*n*_ clusters are also displayed in Fig. 1. Note that the isomers of Mg_*n*_ reported by earlier works^29,30,36–41^ are reproduced by utilizing CALYPSO structural searching method. The most stable structures of the Mg_*n*_ clusters obtained in our searches are consistent with previous results. This suggests the current method is capable of correctly identifying the lowest energy structure of magnesium and magnesium-based clusters. We list the electronic states and point group symmetries of the most stable isomers of Mg_*n*_ and Be_2_Mg_*n*_ in Table 1.

**Table 1:** Electronic states, symmetries, average binding energies *E*_b_ (eV), HOMO-LUMO energy gaps *E*_g_ (eV), and charges on the Be atoms of the most stable Mg_*n*+2_ and Be_2_Mg_*n*_ (*n* = 1–20) clusters.

Clusters	Mg_*n*+2_				Be_2_Mg_*n*_					
	Sta.	Sym.	*E*_b_	*E*_g_	Sta.	Sym.	*E*_b_	*E*_g_	Charge	
									Be-1	Be-2
*n* = 1	^1^∑	*D*_3*h*_	0.129	2.865	^1^A_1_	*C*_2*v*_	0.343	2.403	−0.164	−0.164
*n* = 2	^1^A′	*T*_*d*_	0.302	2.899	^1^A_1_	*C*_2*v*_	0.634	2.582	−0.457	−0.457
*n* = 3	^1^A_1_	*D*_3*h*_	0.301	2.170	^1^A_1_	*C*_2*v*_	0.659	1.921	−0.826	−0.826
*n* = 4	^1^A_1_′	*C*_2*v*_	0.315	1.982	^1^A_1g_	*D*_4*h*_	0.624	1.862	−1.094	−1.094
*n* = 5	^1^A_1_	*D*_5*h*_	0.369	2.089	^1^A′	*C*_*s*_	0.643	1.673	−1.895	−0.630
*n* = 6	^1^A_1_′	*C*_*s*_	0.398	1.777	^1^A′	*C*_*s*_	0.659	1.611	−1.351	−0.766
*n* = 7	^1^A′	*D*_3*h*_	0.485	1.527	^1^A	*C*_1_	0.706	1.312	−0.735	−1.051
*n* = 8	^1^A′	*C*_3*v*_	0.542	1.958	^1^A′	*C*_*s*_	0.797	1.988	−1.738	−1.738
*n* = 9	^1^A_1_	*D*_3*h*_	0.532	2.047	^1^A′	*C*_*s*_	0.765	1.589	−2.494	−1.560
*n* = 10	^1^A_1_′	*C*_*s*_	0.530	1.430	^1^A	*C*_1_	0.776	1.514	−1.427	−2.512
*n* = 11	^1^A′	*C*_*s*_	0.550	1.552	^1^A	*C*_1_	0.787	1.593	−2.626	−1.449
*n* = 12	^1^A′	*C*_1_	0.556	1.212	^1^A	*C*_1_	0.770	1.141	−1.767	−2.416
*n* = 13	^1^A	*D*_3*h*_	0.603	1.219	^1^A_1_	*C*_2*v*_	0.800	1.123	−2.600	−1.925
*n* = 14	^1^A_1_′	*C*_1_	0.602	1.028	^1^A′	*C*_*s*_	0.797	0.904	−2.465	−1.679
*n* = 15	^1^A	*D*_4*d*_	0.660	1.101	^1^A′	*C*_*s*_	0.824	1.187	−2.097	−1.671
*n* = 16	^1^A_1_	*C*_2*v*_	0.680	1.055	^1^A	*C*_1_	0.830	0.934	−1.402	−1.575
*n* = 17	^3^B_2_	*C*_*s*_	0.700	0.988	^1^A	*C*_1_	0.848	0.932	−1.770	−1.894
*n* = 18	^1^A_1_	*C*_3_	0.726	1.523	^1^A	*C*_1_	0.869	1.438	−2.129	−2.085
*n* = 19	^1^A	*C*_1_	0.706	1.460	^1^A	*C*_1_	0.865	1.220	−2.328	−1.850
*n* = 20	^1^A	*C*_1_	0.705	1.291	^1^A	*C*_1_	0.855	1.066	−2.194	−2.256

Let us compare geometries of Be_2_Mg_*n*_ to those of pure magnesium clusters. We can find several attractive results. (1) The transition point from 2D to 3D structures is *n* = 2 for Be_2_Mg_*n*_ clusters, which is consistent with that of pure Mg_*n*+2_ clusters. However, the configurations transition from hollow 3D structures to filled cage-like frameworks emerges at *n* = 10 for Be_2_Mg_*n*_ clusters, which is obviously earlier than that of *n* = 14 for Mg_*n*+2_ clusters. (2) The Be_2_Mg_*n*_ clusters keep the original shapes of the corresponding Mg_*n*+2_ clusters at *n* = 1–3, 5–8 and 16. Namely, the most stable isomers of the Be_2_Mg_1–3,5–8,15_ clusters are viewed as the substituted structures of corresponding Mg_3–5,7–10,17_ clusters, in which two Mg atoms are replaced by Be atoms. Due to the difference of atomic radius of Mg and Be atoms, these doped structures occur with some degree of distortion. Conversely, the remaining Be_2_Mg_*n*_ clusters show different geometries in contrast to pure Mg_*n*+2_ clusters. It implies that the dopants might produce major influence on the frameworks of magnesium clusters in these case. (3) The Be impurity atoms prefer the convex capped sites in the size range of *n* = 1–4, then trend to the surface sites of the skeleton in clusters size of *n* = 5–9. As the number of Mg increases, stating from *n*–10, a different trend emerges. One of the Be atoms still localizes at the surface site, while the other tend to embed itself inside the magnesium motif. In Be_2_Mg_16–18_ clusters, the Be atom is back at the surface sites of the skeleton. At the number of Mg larger than eighteen, the Be impurity atoms have been completely encapsulated by caged magnesium framework and made more Be-Mg bonds together with surrounding Mg atoms. (4) It is worth mentioning that the most stable isomer of Mg_17_ cluster displays a fascinating cage structure with high *D*_4*d*_ symmetry. Although the Be_2_Mg_15_ cluster remains this cage structure, the symmetry turns to *C*_*s*_ from *D*_4*d*_ owing to effect of dopants. As shown in Fig. S2, we have predicted a high symmetric Be_2_Mg_15_ cluster (*D*_5*h*_ symmetry) with a pentagram-shaped structure, however, it is 0.16 eV higher in total energy than the most stable isomer of Be_2_Mg_15_ cluster. In fact, for Be_2_Mg_16–18_ clusters, we also have generated some low-lying structures with two Be atoms being completely encapsulated. Further energy calculation show that these structures are less stable than the corresponding most stable isomers. Similarly, some low-lying structures with two Be atoms localizing at the surface site have been yielded for Be_2_Mg_19–20_ clusters. The present results suggest that the isomers are all less stable.

### Size dependence of relative stability

**Figure 2:**
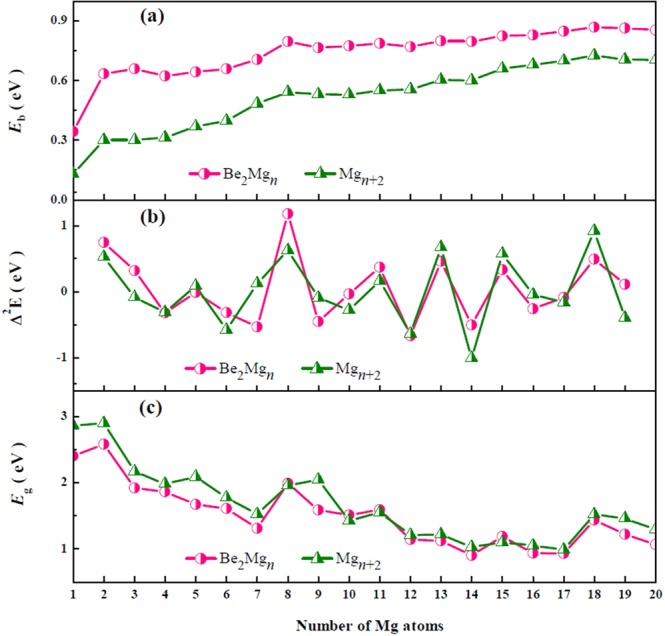
Size dependence of (a) the average binding energy ${E}_{b}$, (b) second-order difference of energy ${\varDelta }^{2}E$, and (c) HOMO-LUMO energy gaps ${E}_{g}$ for Mg_*n*+2_ and Be_2_Mg_*n*_ (*n* = 1–20) clusters.

Studies of the average binding energy (${E}_{b}$) can, therefore, shed light on the size-induced inherent stability of the Mg_*n*+2_ and Be_2_Mg_*n*_ (*n* = 1–20) clusters. For mentioned clusters, ${E}_{b}$ values are calculated as

1 $${E}_{b}({{\rm{Mg}}}_{n})=\frac{nE({\rm{Mg}})-E({{\rm{Mg}}}_{n})}{n},$$

2 $${E}_{b}({{\rm{Be}}}_{2}{{\rm{Mg}}}_{n})=\frac{nE({\rm{Mg}})+2E({\rm{Be}})-E({{\rm{Be}}}_{2}{{\rm{Mg}}}_{n})}{n+2},$$

where $E$ denote the total energy of the corresponding clusters or atoms. Figure 2(a) shows how the ${E}_{b}$ values of the Mg_*n*+2_ and Be_2_Mg_*n*_ clusters evolve with increasing cluster size. The features of the curves are easily explained, as are the local maximum of the shapes corresponding to those clusters with robust stabilities. In the size range studied here, the ${E}_{b}$ values of doped species are always much higher than those of pure clusters. It suggests that the stabilities of the pure magnesium clusters can be enhanced by means of Be atoms doping. As cluster size increases, the averaged binding energies of doped clusters approach gradually those of pure magnesium clusters because the contributing proportion of Be atoms decreases in larger size doped clusters. The graphics of ${E}_{b}$ for both Mg_*n*+2_ and Be_2_Mg_*n*_ clusters show the similar pattern, namely generally growing behavior with increase of cluster size and common local maximum at *n* = 2, 8, and 18. For large clusters, *n* ≥ 19, the values of ${E}_{b}$ decrease somewhat. The appearance of the maximum indicates that Be_2_Mg_2,8,18_ and Mg_4,10,20_ clusters have relatively strong energetic stabilities. This finding can be interpreted by spherical Jellium model, in which the spherical magic clusters with 8, 20, 40 valence electrons corresponding to the closed electronic subshells turn out to be relatively stable.

Another manifestation of the size-induced variation in cluster stability is exhibited in Fig. 2(b), which depicts the dependence of the second-order energy difference (${\varDelta }^{2}E$) on cluster size. For mentioned clusters, ${\varDelta }^{2}E$ values are calculated as

3 $${\varDelta }^{2}E({{\rm{Mg}}}_{n})=E({{\rm{Mg}}}_{n-1})+E({{\rm{Mg}}}_{n+1})-2E({{\rm{Mg}}}_{n}),$$

4 $${\varDelta }^{2}E({{\rm{Be}}}_{2}{{\rm{Mg}}}_{n})=E({{\rm{Be}}}_{2}{{\rm{Mg}}}_{n-1})+E({{\rm{Be}}}_{2}{{\rm{Mg}}}_{n+1})-2E({{\rm{Be}}}_{2}{{\rm{Mg}}}_{n}).$$

As shown in the graphics, for both the Mg_*n*+2_ and Be_2_Mg_*n*_ clusters, the ${\varDelta }^{2}E$ display the similar odd-even oscillating behaviors in the range of *n* = 10 − 16. There are several obvious peaks at *n* = 5, 8, 11, 13, 15, and 18. It can reflect more relative stabilities of the Be_2_Mg_*n*_ and Mg_*n*+2_ (*n* = 5, 8, 11, 13, 15, and 18) clusters. Specially, the Be_2_Mg_8_ cluster has the largest ${\varDelta }^{2}E$ value of 1.17 eV.

Let us now regard the highest occupied and the lowest unoccupied molecular orbitals energy gaps (HOMO–LUMO gaps) of the mentioned clusters. According to the energies of the frontier molecular orbitals, gaps ${E}_{g}$ of the most stable isomers of the Mg_*n*+2_ and Be_2_Mg_*n*_ (*n* = 1–20) cluster are calculated and listed in Table 1. Meanwhile, the change of ${E}_{g}$ *versus* the number of Mg atoms are shown in Fig. 2(c). One can find that the values of ${E}_{g}$ for them exhibits a slowly but rather nonmonotonously decreasing tendency, indicating the more enhancive metallicities for the larger-sized Mg_*n*+2_ and Be_2_Mg_*n*_ clusters. It is worth noting that tendency of gaps for pure magnesium clusters agree well with previous findings by Kuang *et al*.^30^, Acioli *et al*.^36,37^, and Lyalin *et al*.^38^. For Be_2_Mg_*n*_ (*n* = 1–20) clusters, the local peaks in the curve of ${E}_{g}$ at *n* = 2, 8, 11, 15, and 18 accord with above analysis based on ${E}_{b}$ and ${\varDelta }^{2}E$. It signifies that these clusters possess dramatically enhanced chemical stability. Furthermore, we come to conclusion that the effect of doping Be atoms on the gaps of the Mg_*n*_ clusters is slight in the range of *n* = 1 − 20. The reason may be that there is similar valence electron structure for Be and Mg atoms.

### Ionization potential, electron affinity, chemical hardness, and charge transfer

**Figure 3:**
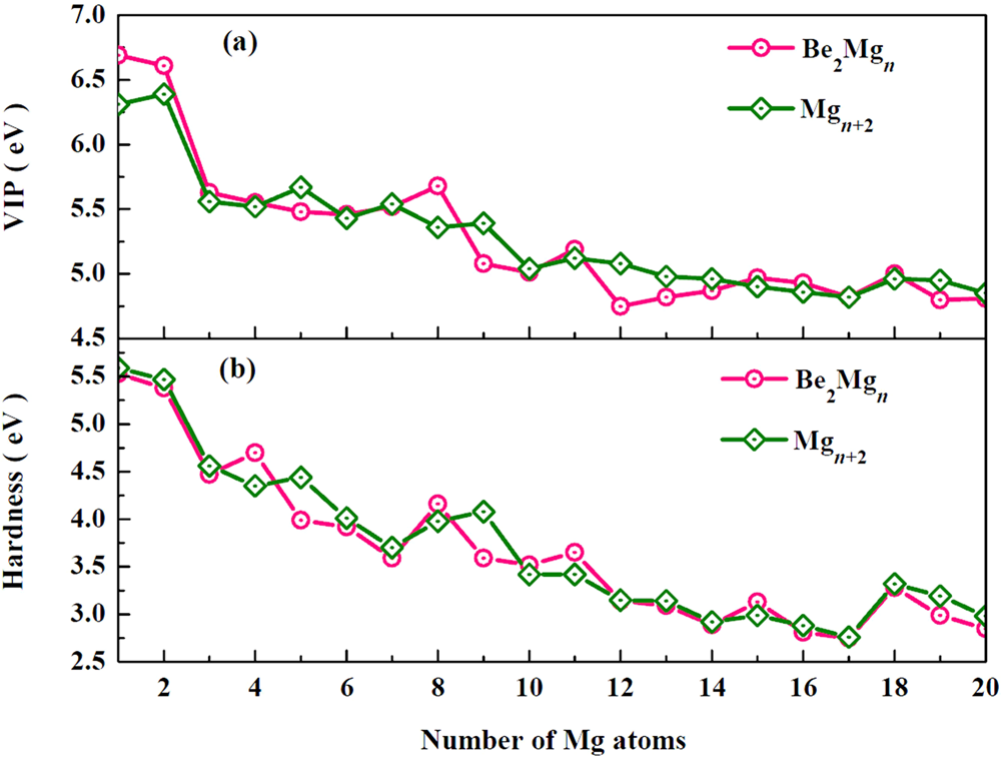
Size dependence of (a) VIP and (b) chemical hardness for Mg_*n*+2_ and Be_2_Mg_*n*_ (*n* = 1–20) clusters.

It is well known that the vertical ionization potential (VIP), vertical electron affinity (VEA), and chemical hardness is very sensitive to the electronic structure of clusters. The correctness of our calculation is supported by the good agreement between experimental and theoretical VIP for the Mg atom (7.646 eV^36^ vs 7.542 eV). In Table 2, we summarize the VIP, VEA, and chemical hardness (*η*) of the Mg_*n*+2_ and Be_2_Mg_*n*_ (*n* = 1–20) clusters. The size-dependent behavior of the VIP is shown in the Fig. 3(a). As the cluster size increases, the curves of VIP for both Mg_*n*+2_ and Be_2_Mg_*n*_ clusters exhibit decreasing tendency in general, which means that it is easier for the larger-sized clusters to lose an electron than the smaller sized ones. On the whole curve of Be_2_Mg_*n*_ clusters, we can clearly see some pronounced local peaks at *n* = 2, 8, 11, 15, and 18, indicating their high chemical stability. Indeed this finding is supported by the results of the chemical hardness. As shown in Fig. 3(b), one can also find the identical local peaks of chemical hardness for Be_2_Mg_2,8,11,15,18_ clusters. It is worth pointing that local maxima of VIP and *η* agree well with the results of the HOMO–LUMO gaps. In addition, the values of *η* for both Mg_*n*+2_ and Be_2_Mg_*n*_ clusters tend to decrease with the growing of the number of Mg atoms for the given size.

**Table 2:** Calculated VIP (eV), VEA (eV), and chemical hardness (*η*, in eV) values of the most stable Mg_*n*+2_ and Be_2_Mg_*n*_ (*n* = 1–20) clusters.

	Mg_*n* + 2_			Be_2_Mg_*n*_		
	VIP	VAE	*η*	VIP	VEA	*η*
*n* = 1	6.31	0.72	5.59	6.69	1.16	5.53
*n* = 2	6.39	0.92	5.47	6.61	1.22	5.38
*n* = 3	5.56	1.00	4.56	5.63	1.16	4.47
*n* = 4	5.52	1.17	4.35	5.55	0.85	4.70
*n* = 5	5.67	1.23	4.44	5.48	1.49	3.99
*n* = 6	5.43	1.42	4.01	5.46	1.54	3.92
*n* = 7	5.54	1.84	3.70	5.52	1.93	3.59
*n* = 8	5.36	1.38	3.98	5.68	1.52	4.16
*n* = 9	5.39	1.31	4.08	5.08	1.49	3.59
*n* = 10	5.04	1.62	3.42	5.01	1.50	3.52
*n* = 11	5.12	1.70	3.42	5.19	1.54	3.65
*n* = 12	5.08	1.93	3.15	4.75	1.61	3.14
*n* = 13	4.98	1.84	3.14	4.82	1.73	3.09
*n* = 14	4.96	2.04	2.92	4.87	1.98	2.89
*n* = 15	4.90	1.91	2.99	4.97	1.84	3.13
*n* = 16	4.86	1.98	2.88	4.93	2.12	2.81
*n* = 17	4.82	2.06	2.76	4.82	2.07	2.75
*n* = 18	4.96	1.64	3.32	5.00	1.71	3.28
*n* = 19	4.95	1.76	3.19	4.80	1.81	2.99
*n* = 20	4.85	1.87	2.98	4.81	1.96	2.85

**Figure 4:**
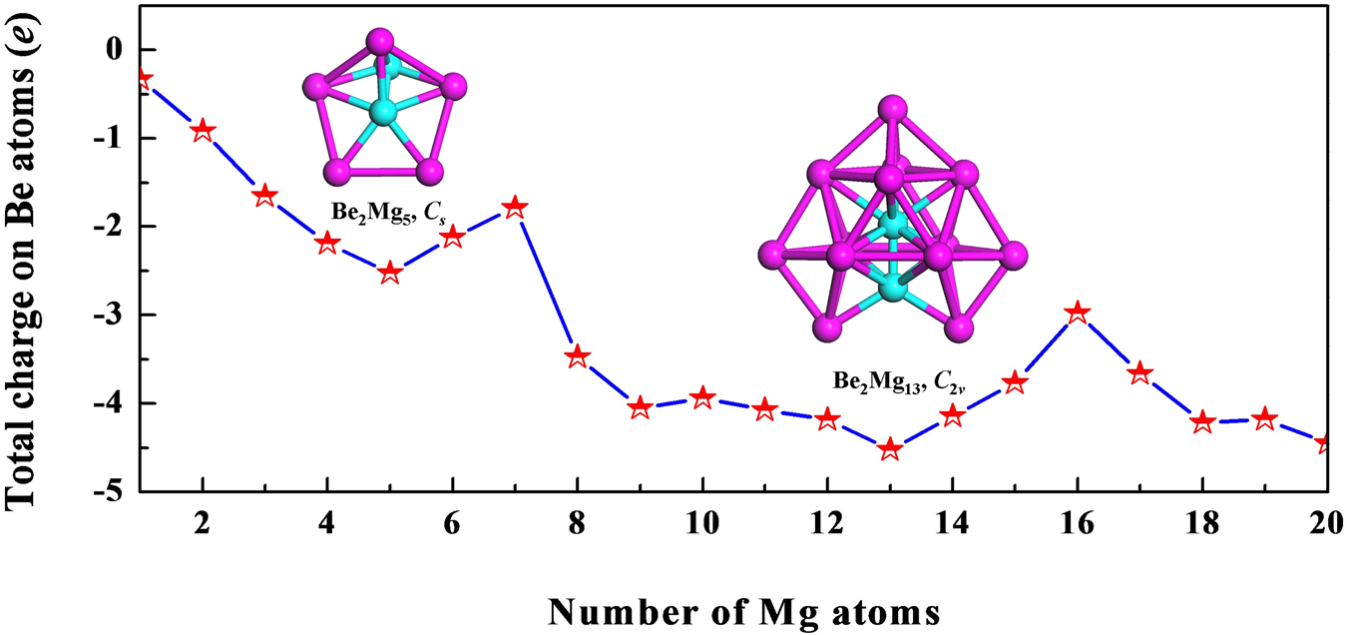
Size dependence of total charge on Be atoms for Be_2_Mg_*n*_ (*n* = 1–20) clusters.

With the aid of the natural population analysis (NPA), we can obtain the charges on Be atoms of the Be_2_Mg_*n*_ (*n* = 1–20) clusters, as listed in Table 1. The results show that the Be atoms in all doped clusters act as the electron acceptors owing to the negative values of charges on them. Since the Be atom (1.59) is much more in electronegativity than the Mg atom (1.31), the partial charge transfer from Mg to Be takes place in Be_2_Mg_*n*_ clusters. In Fig. 4, we depict the size dependence of total charge on Be atoms of the Be_2_Mg_*n*_ clusters. The results show that charge transfer, in general, increases with the increasing cluster size. Two visible maximum of charge transfer occur at *n* = 5 and 13. To extract more charge transfer information, the natural electronic configuration (NEC) of the Be_2_Mg_*n*_ (*n* = 1–20) clusters are listed in Table S1 of supplementary information. From the table, it is found that there is a large donation of electrons from the Be-2*s* and Mg-3*s* orbitals. Meanwhile, in corresponding clusters, the unfilled Be-2*p* orbitals accept 0.63–3.76 electrons, while Mg-3*p* orbitals accept 0.10–2.09 electrons, which illustrates the charge transfers occur in intramural *s* and *p* orbitals of the clusters. As the cluster size increases, the occupations of the Be-2*s* and Mg-3*s* orbitals decease while their 2*p* and 3*p* orbitals occupations increase. Previous works on Mg_*n*_ clusters^36,37^ have proposed that the occupations of *p* orbital in Mg_*n*_ clusters is an evident character of metallic bonding. Therefore, an increase in the occupations of the Be-2*p* and Mg-3*p* orbitals is a marker of the increasing metallic behavior of Be_2_Mg_*n*_ clusters.

## Bonding Characters

**Figure 5:**
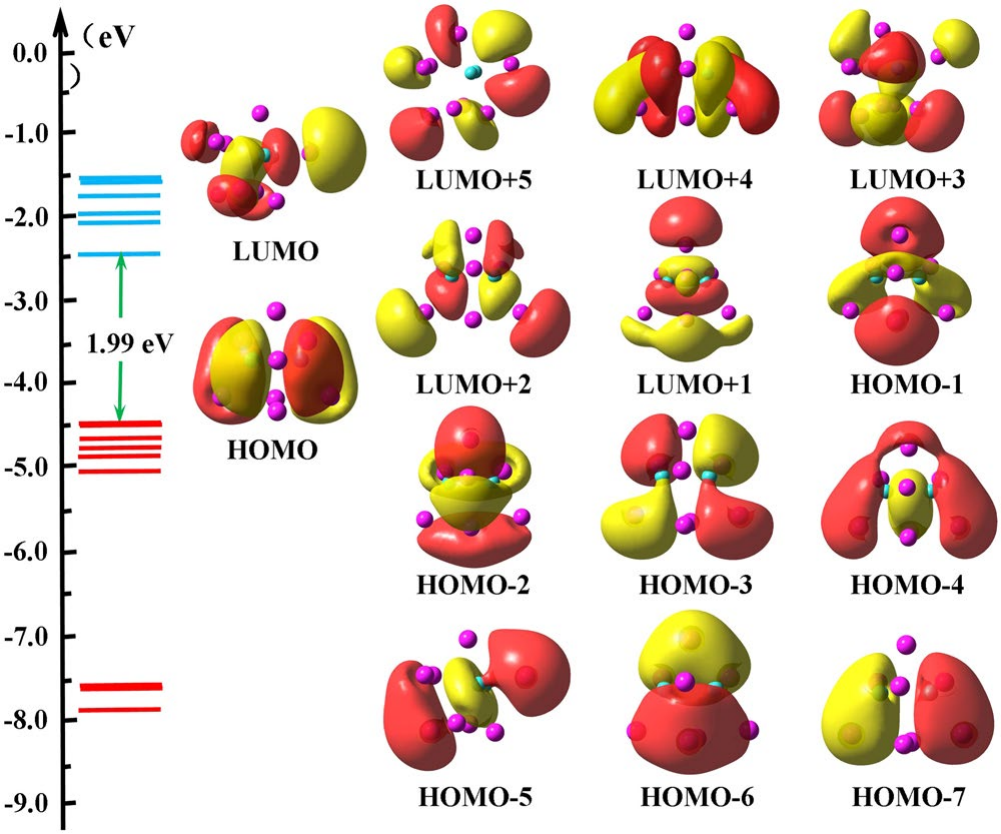
Molecular orbitals and the corresponding energy levels of the Be_2_Mg_8_ cluster. The HOMO-LUMO gap is indicated (in green).

It would be interesting to shed light on the chemical bonding properties of the Be_2_Mg_*n*_ clusters. Due to the outstanding stability, we select Be_2_Mg_8_ to be a acceptable candidate for probing the bonding feature. In Fig. 5, we analyzed its molecular orbitals and the corresponding energy levels. Because the Be_2_Mg_8_ cluster has a shell-closed electronic structure, the HOMO is doubly occupied and the LUMO is 1.99 eV higher in energy than the HOMO. Based on natural bond orbital (NBO) analysis, the components of the given molecular orbital can be obtained by Multiwfn program. For Be_2_Mg_8_ cluster, the components of LUMO contain the 13.4% of *p* atomic orbital (AO) of two Be atoms as well as 37.5% of *s* AO and 49.1% of *p* AO from Mg atoms. The HOMO is generated by 21.6% of Be-*s* AO and 25.2% of Be-*p* AO along with 53.2% Mg-*s* and Mg-*p* AO. For other occupied MOs, the *s* orbitals of the Be atoms have 6.4% contribution to the HOMO-1, while *s* and *p* AO of Mg atoms have 93.6% contribution to it. The *p*_*x*_ and *p*_*y*_ orbitals of Be atoms as well as the Mg-*s*, Mg-*p*_*x*_, and Mg-*p*_*y*_, are mainly included in HOMO-2 and HOMO-6. The HOMO-3 and HOMO-5 mostly originate from Be-*p*, Mg-*s*, and Mg-*p* AOs. There are 37% of Be-*p*_*z*_, 42.9% of Mg-*s*, and 20% Mg-*p* AO in HOMO-4. With regard to HOMO-7, the molecular orbital is mainly formed by equivalent contributions (about 23%) of Be-*s* and Be-*p* together with 33% Mg-*s* and 21% Mg-*p*_*z*_ AOs. It is remarkable that the *s-p* hybridization between the Be and Mg atoms might lead to strong Be-Mg bonds in Be_2_Mg_8_ cluster.

**Figure 6:**
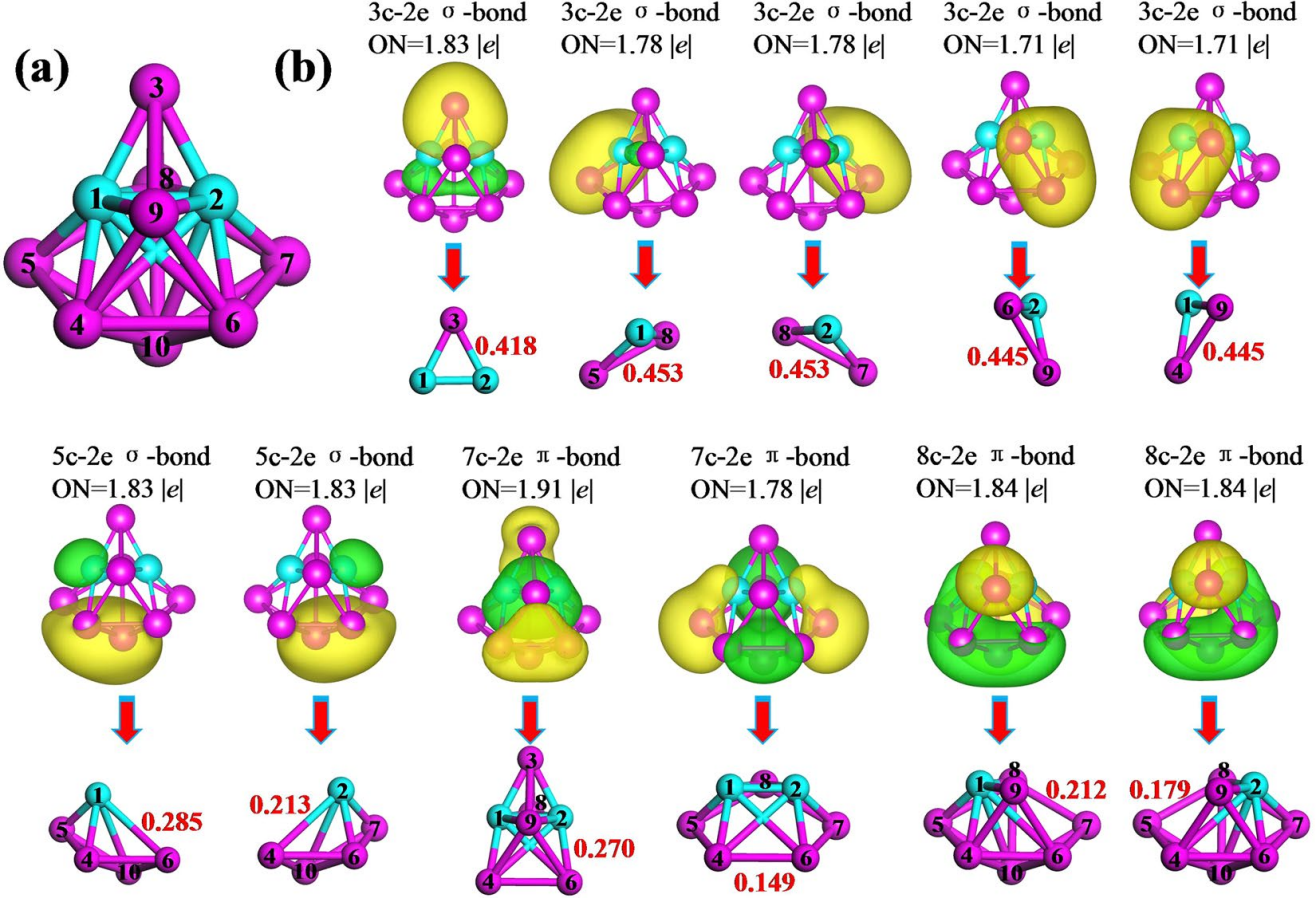
Analysis of (a) the structural diagram with all atomic labels and (b) AdNDP chemical bonds and multi-center bond orders for the Be_2_Mg_8_ cluster. ON denotes the occupation number.

To further understanding the bonding characters, we analyze the multi-center bonds and corresponding bond orders. Based on the AdNDP method, a systemic search of *n*-center two-electron (*n*c-2*e*) bonds is performed. It is a localized bond when *n* = 1 and 2, whereas it is delocalized bond when *n* ≥ 3. The most stable Be_2_Mg_8_ cluster is to be a suitable candidate. To give a clear insight of the distribution of the multi-center bonds, we first provide a structural diagram with all atomic labels in Fig. 6(a). Then the Fig. 6(b) displays the multi-center bonds and their occupation numbers (ONs) of Be_2_Mg_8_ cluster. Below each multi-center bonds images, the structural units generated these multi-center bonds and their bond orders are exhibited. The theoretical results reveal that non localized bond, however, eleven delocalized bonds are included in Be_2_Mg_8_ cluster. In detail, one 3c-2*e* σ-type bond with ON = 1.83 |*e*| is formed by trigonal Be(1)-Be(2)-Mg(3) unit. The multi-center bond order is 0.418. Four delocalized bonds are all 3c-2e σ-type bonds (ON = 1.78–1.71 |*e*|) with contributions of each planar BeMg_2_ units. Because of different Be-Mg bond lengths in different units, it is found that the multi-center bond orders are the highest value of 0.453 for BeMg_2_ units including atom labels of 1–5–8 and 2–7–8, whereas they are 0.445 for 2–6–9 and 1–4–9 of BeMg_2_ units. There are two 5c-2e σ-type bonds with ON = 1.83 |*e*|, which is derived from two BeMg_4_ units of 1-4-5-6-10 and 2-4-6-7-10. Their multi-center bond orders are 0.285 and 0.213, respectively. Two 7c-2e with ON = 1.91–1.78 |*e*| (two different Be_2_Mg_5_ units) and two 8c-2e with ON = 1.84 |*e*| show π-type bonding character. Their multi-center bond orders are 0.272, 0.149, 0.212, and 0.179, respectively. In order to deeply examine the nature of bonding, the Mayer bond orders for each bonds of the most stable Be_2_Mg_8_ cluster are listed in Table S2. From the table, we can find that the Be atoms have formed some strong Be-Mg bonds together with their adjacent Mg atoms because the Mayer bond orders of these Mg-Be bonds are bigger than those of Be-Be bond and the most Mg-Mg bonds in Be_2_Mg_8_ cluster.

## Conclusions

In a summary, for the most stable Be_2_Mg_*n*_ clusters, the transition point from 2D to 3D structures is *n* = 2 and configurations transition from hollow 3D structures to filled cage-like frameworks emerges at *n* = 10. In small sized, two Be atoms prefer surface capped positions. Then one Be atom tends to embed itself inside the magnesium motif with increasing cluster size. When *n* ≥ 19, two Be atoms have been completely encapsulated by caged magnesium framework. It is found that the charges transfer from Mg to Be atoms in all Be_2_Mg_*n*_ clusters. As the cluster size grows, the occupations of the Be-2*p* and Mg-3*p* orbitals increase, indicating the increasing metallic behavior of Be_2_Mg_*n*_ clusters. The investigation of the clusters stability reveal that the stabilities of Mg_*n*_ clusters can be enhanced by means of Be atoms doping and Be_2_Mg_8_ cluster is very stable in studied cluster range. There are obvious *s*-*p* hybridization of the Be and Mg atoms in Be_2_Mg_8_ cluster, which might induce stronger Be-Mg bonds. It is supported by the multi-center bonds and Mayer bond order analysis.

### Computational methods

To achieve the the most stable isomers of Mg_*n*+2_ and Be_2_Mg_*n*_ clusters, it is necessary to utilize an efficient algorithm of global optimization for searching the global minima of the different sized clusters. Here we rely on the CALYPSO code^45–47^, which can quickly search the most stable structures of the clusters on the bases of the particle swarm optimization algorithm. The validity of this method for clusters structure predicting has been demonstrated by its successful application of various clusters^48–55^. In detail, trial structures of the clusters are ordered in generations in the process of searching. Each generation contains 50 structures, 60% of which are produced by PSO algorithm, whereas the rest is generated randomly. We performed 30 generations to produce 1000–1500 structurally different isomers for each clusters. The underlying geometric optimizations and energy computations for these isomers are performed at B3PW91/6–31 G level^56–58^. Among them, the isomers whose total energies fell into the 3 eV interval comparison with the lowest energy are reoptimized along with frequency calculation at B3PW91/6–311 G(d, p) level^58^, as implemented in the Gaussian 09 program package^59^. The effect of spin multiplicity (up to septet) is taken into account in optimized procedure. It is worth pointing out the most stable isomers are found to prefer the singlet spin state, except for Mg_18_ cluster (triplet spin state). Based on Multiwfn program^60^, the chemical bonding characters of the Be_2_Mg_*n*_ clusters have been analyzed by utilizing the adaptive natural density partitioning (AdNDP) method^61^.

## Supplementary information

Supplementary Information.
