# Supplementary material for: Probing the structural evolution and electronic properties of divalent metal Be2Mgn clusters from small to medium-size
Source: Sci Rep. 2020 Apr 8;10:6052. doi: 10.1038/s41598-020-63237-8 (PMC7142069; doi:10.1038/s41598-020-63237-8)
Supplement: Supplementary file 1 — Supplementary Information. [file 41598_2020_63237_MOESM1_ESM.pdf]

Support information

Probing the structural evolution and electronic properties of divalent metal Be_2Mg_n clusters from small to medium-size

Feige Zhang [1], Hairong Zhang [1], Wang Xin [2], Peng Chen [2], Yanfei Hu [3], Xiaoyi Zhang [2] & Yaru Zhao [2,*]

[1] School of Electrical and Electronic Engineering, Baoji University of Arts and Sciences, Baoji 721016, China

[2] College of Physics and Optoelectronics Technology, Baoji University of Arts and Sciences, Baoji 721016, China

[3] School of Physics and Electronic Engineering, Sichuan University of Science & Engineering, Zigong 643000, China

[*]Correspondence author. E-mail: bjwl_zyr@163.com (Y. R. Zhao)

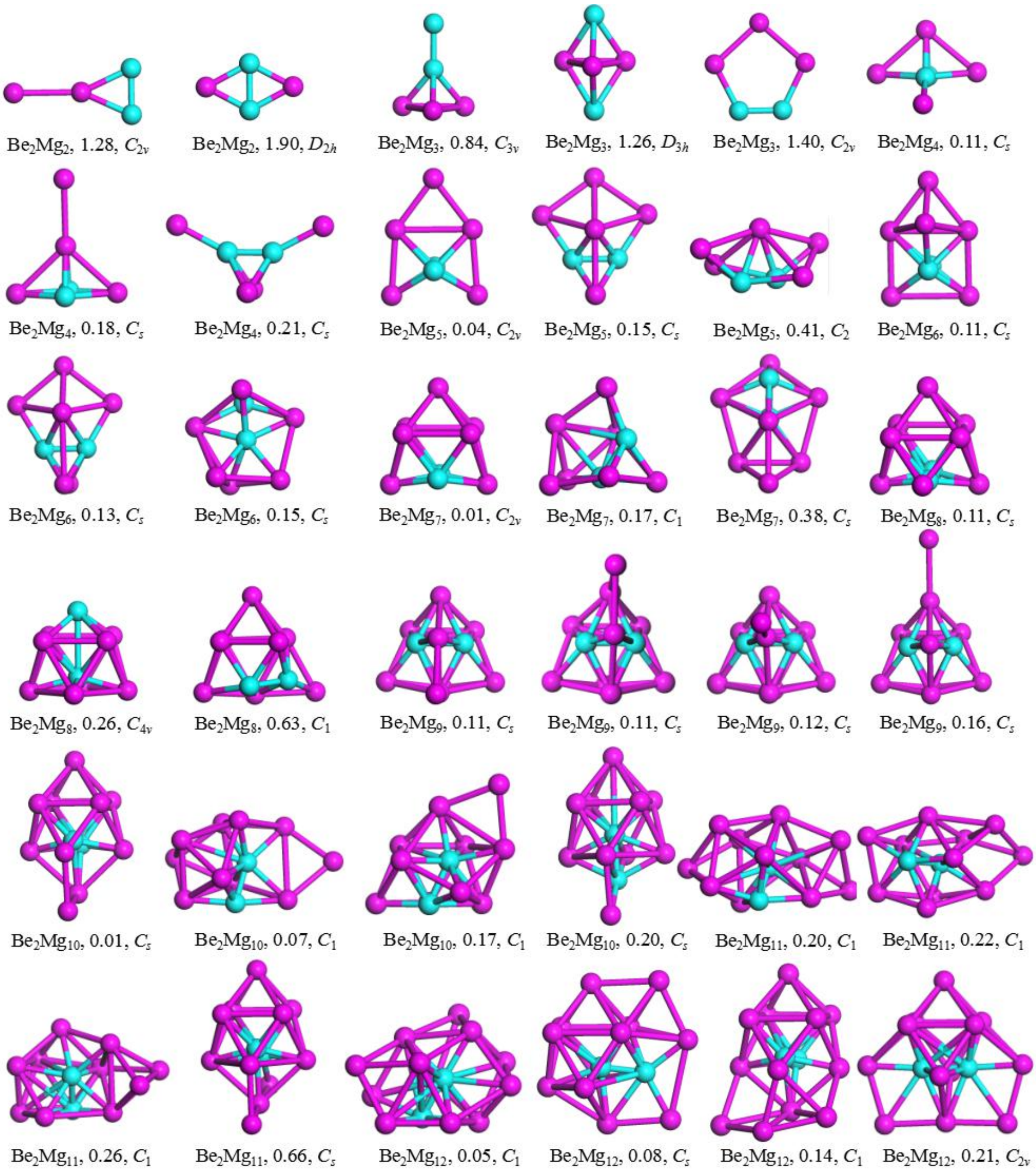

Fig. S1 The typical low-lying isomers with their relative energies (eV) and symmetries of the Be_2Mg_n (n=1–12) clusters.

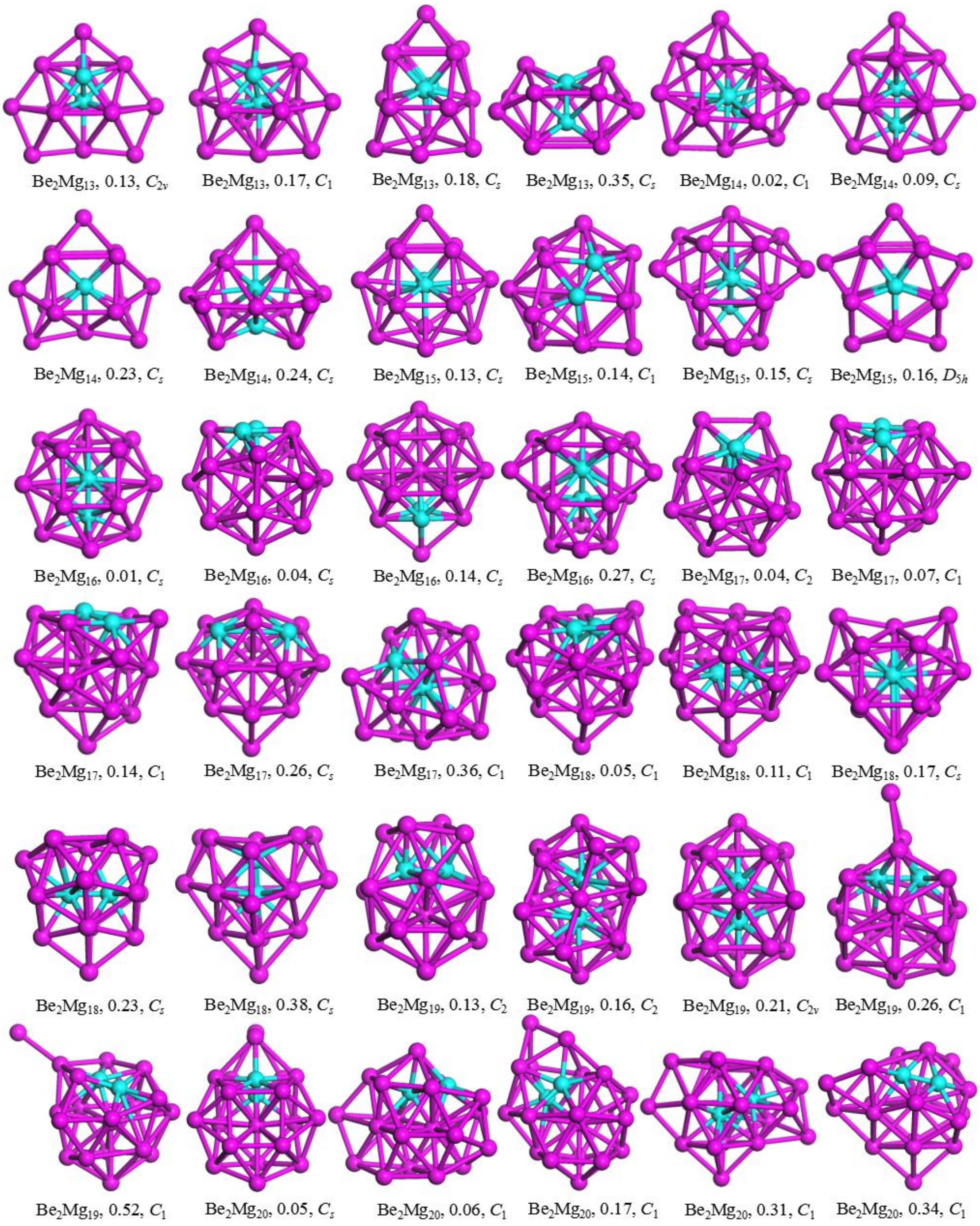

Fig. S2 The typical low-lying isomers with their relative energies (eV) and symmetries of the Be_2Mg_n (n=13–20) clusters.

Table S1 The natural electronic configuration (NEC) of the Be_2Mg_n (n=1–20) clusters

Clusters	NEC		
	Be (1)	Be(2)	Mg
Be_2Mg	$2s^{1.52}2p^{0.63}3p^{0.01}$	$2s^{1.52}2p^{0.63}3p^{0.01}$	$3s^{1.43}3p^{0.24}$
Be_2Mg_2	$2s^{1.22}2p^{1.22}3p^{0.02}$	$2s^{1.22}2p^{1.22}3p^{0.02}$	$3s^{1.12}3p^{0.42}4p^{0.01}$
Be_2Mg_3	$2s^{1.15}2p^{1.64}3p^{0.03}$	$2s^{1.15}2p^{1.64}3p^{0.03}$	$3s^{1.02}3p^{0.31}4p^{0.01}$
Be_2Mg_4	$2s^{1.16}2p^{1.89}3p^{0.03}$	$2s^{1.16}2p^{1.89}3p^{0.03}$	$3s^{1.26}3p^{0.19}$
Be_2Mg_5	$2s^{1.16}2p^{2.72}3p^{0.01}$	$2s^{1.02}2p^{1.59}3p^{0.02}$	$3s^{0.93\text{-}1.02}3p^{0.34\text{-}0.47}4p^{0.01}$
Be_2Mg_6	$2s^{1.07}2p^{2.24}3p^{0.01}$	$2s^{0.96}2p^{1.78}3p^{0.02}$	$3s^{0.87\text{-}1.21}3p^{0.34\text{-}0.71}4p^{0.01}$
Be_2Mg_7	$2s^{1.03}2p^{1.68}3p^{0.02}$	$2s^{0.99}2p^{2.02}3p^{0.02}$	$3s^{0.90\text{-}1.18}3p^{0.59\text{-}0.83}4p^{0.01\text{-}0.02}$
Be_2Mg_8	$2s^{1.05}2p^{2.67}3p^{0.01}$	$2s^{1.05}2p^{2.67}3p^{0.01}$	$3s^{0.83\text{-}1.14}3p^{0.29\text{-}0.83}4p^{0.01\text{-}0.02}$
Be_2Mg_9	$2s^{0.95}2p^{3.52}3p^{0.02}$	$2s^{0.91}2p^{2.63}3p^{0.01}$	$3s^{0.79\text{-}1.25}3p^{0.10\text{-}0.85}4p^{0.01\text{-}0.02}$
Be_2Mg_{10}	$2s^{0.94}2p^{2.47}3p^{0.01}$	$2s^{0.96}2p^{3.54}3p^{0.01}$	$3s^{0.80\text{-}1.30}3p^{0.32\text{-}0.79}4p^{0.01\text{-}0.02}$
Be_2Mg_{11}	$2s^{0.92}2p^{3.69}3p^{0.01}$	$2s^{0.94}2p^{2.49}3p^{0.02}$	$3s^{0.78\text{-}1.17}3p^{0.57\text{-}0.74}4p^{0.01\text{-}0.02}$
Be_2Mg_{12}	$2s^{0.99}2p^{2.76}3p^{0.02}$	$2s^{0.92}2p^{3.48}3p^{0.02}$	$3s^{0.73\text{-}1.10}3p^{0.56\text{-}1.01}4p^{0.01\text{-}0.02}$
Be_2Mg_{13}	$2s^{0.82}2p^{3.76}3p^{0.02}$	$2s^{0.86}2p^{3.04}3p^{0.02}$	$3s^{0.78\text{-}1.09}3p^{0.45\text{-}0.95}4p^{0.01\text{-}0.02}$
Be_2Mg_{14}	$2s^{0.84}2p^{3.60}3p^{0.02}$	$2s^{0.88}2p^{2.78}3p^{0.02}$	$3s^{0.73\text{-}1.00}3p^{0.53\text{-}1.02}4p^{0.01\text{-}0.02}$
Be_2Mg_{15}	$2s^{0.81}2p^{3.25}3p^{0.04}$	$2s^{0.88}2p^{2.77}3p^{0.02}$	$3s^{0.71\text{-}0.99}3p^{0.62\text{-}1.09}4p^{0.01\text{-}0.02}$
Be_2Mg_{16}	$2s^{0.85}2p^{2.53}3p^{0.01}$	$2s^{0.87}2p^{2.69}3p^{0.02}$	$3s^{0.58\text{-}1.05}3p^{0.59\text{-}2.09}4p^{0.01\text{-}0.02}$
Be_2Mg_{17}	$2s^{0.84}2p^{2.91}3p^{0.01}$	$2s^{0.90}2p^{2.98}3p^{0.01}$	$3s^{0.61\text{-}0.93}3p^{0.33\text{-}1.99}4p^{0.01\text{-}0.02}$
Be_2Mg_{18}	$2s^{0.88}2p^{3.24}3p^{0.01}$	$2s^{0.84}2p^{3.23}3p^{0.01}$	$3s^{0.59\text{-}0.93}3p^{0.41\text{-}1.83}4p^{0.01\text{-}0.02}$
Be_2Mg_{19}	$2s^{0.80}2p^{3.50}3p^{0.03}$	$2s^{0.71}2p^{3.10}3p^{0.04}$	$3s^{0.75\text{-}0.93}3p^{0.60\text{-}1.31}4p^{0.01\text{-}0.02}$
Be_2Mg_{20}	$2s^{0.79}2p^{3.39}3p^{0.01}$	$2s^{0.80}2p^{3.43}3p^{0.02}$	$3s^{0.62\text{-}1.09}3p^{0.51\text{-}1.97}4p^{0.01\text{-}0.02}$

Table S2 The Mayer bond orders of the Be_2Mg_8 clusters

Atoms	Be-1	Be-2	Mg-3	Mg-4	Mg-5	Mg-6	Mg-7	Mg-8	Mg-9
Be-2	0.336								
Mg-3	0.504	0.504							
Mg-4	0.288	0.090	0.114						
Mg-5	0.444	0.135	0.075	0.427					
Mg-6	0.090	0.288	0.114	0.333	0.087				
Mg-7	0.135	0.444	0.075	0.057	0.072	0.427			
Mg-8	0.283	0.283	0.334	0.126	0.393	0.126	0.393		
Mg-9	0.388	0.388	0.230	0.381	0.096	0.381	0.096	0.187	
Mg-10	0.092	0.092	0.118	0.287	0.395	0.287	0.395	0.258	0.093
